# A Structure-function Analysis of Hepatocyte Arginase 2 Reveals Mitochondrial Ureahydrolysis as a Determinant of Glucose Oxidation

**DOI:** 10.1016/j.jcmgh.2024.01.016

**Published:** 2024-01-25

**Authors:** Yiming Zhang, Jiameng Sun, Henry D. Wasserman, Joshua A. Adams, Cassandra B. Higgins, Shannon C. Kelly, Louise Lantier, Brian J. DeBosch

**Affiliations:** 1 Department of Pediatrics, Washington University School of Medicine, St. Louis, Missouri; 2 Vanderbilt Mouse Metabolic Phenotyping Center, Nashville, Tennessee; 3 Department of Cell Biology and Physiology, Washington University School of Medicine, St. Louis, Missouri

**Keywords:** Arginase, Diabetes, Energy Metabolism, Glucose Transport, GLUT, Insulin Resistance, Metabolic Dysfunction-Associated Steatotic Liver Disease, Mitochondria, Obesity, Oxidation, Proteomics, Thermogenesis, Urea Cycle

## Abstract

**Background & Aims:**

Restoring hepatic and peripheral insulin sensitivity is critical to prevent or reverse metabolic syndrome and type 2 diabetes. Glucose homeostasis comprises in part the complex regulation of hepatic glucose production and insulin-mediated glucose uptake and oxidation in peripheral tissues. We previously identified hepatocyte arginase 2 (Arg2) as an inducible ureahydrolase that improves glucose homeostasis and enhances glucose oxidation in multiple obese, insulin-resistant models. We therefore examined structure-function determinants through which hepatocyte Arg2 governs systemic insulin action and glucose oxidation.

**Methods:**

To do this, we generated mice expressing wild-type murine Arg2, enzymatically inactive Arg2 (Arg2^H160F^) and Arg2 lacking its putative mitochondrial targeting sequence (Arg2^Δ1-22^). We expressed these hepatocyte-specific constructs in obese, diabetic (*db/db*) mice and performed genetic complementation analyses in hepatocyte-specific Arg2-deficent (Arg2^LKO^) mice.

**Results:**

We show that Arg2 attenuates hepatic steatosis, independent of mitochondrial localization or ureahydrolase activity, and that enzymatic arginase activity is dispensable for Arg2 to augment total body energy expenditure. In contrast, mitochondrial localization and ureahydrolase activity were required for Arg2-mediated reductions in fasting glucose and insulin resistance indices. Mechanistically, Arg2^Δ1-22^ and Arg2^H160F^ failed to suppress glucose appearance during hyperinsulinemic-euglycemic clamping. Quantification of heavy-isotope-labeled glucose oxidation further revealed that mistargeting or ablating Arg2 enzymatic function abrogates Arg2-induced peripheral glucose oxidation.

**Conclusion:**

We conclude that the metabolic effects of Arg2 extend beyond its enzymatic activity, yet hepatocyte mitochondrial ureahydrolysis drives hepatic and peripheral oxidative metabolism. The data define a structure-based mechanism mediating hepatocyte Arg2 function and nominate hepatocyte mitochondrial ureahydrolysis as a key determinant of glucose oxidative capacity in mammals.

## Graphical abstract

**Figure:**
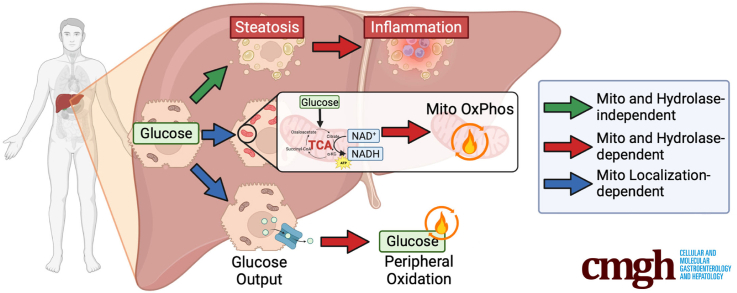

**Summary**

The arginases comprise 2 primary isoforms, which hydrolyze arginine to ornithine and urea. This work identifies hepatocyte mitochondrial ureahydrolysis via arginase 2 as a key driver of hepatic and peripheral oxidative metabolism, which can be leveraged to treat metabolic disease.

Obesity and insulin resistance are the physiological manifestation of deranged glucose and energy homeostasis. Positioned at the nexus of portal and systemic circulations, the hepatocyte is among the primary sensors for systemic glucose and energy homeostasis in response to the acute fed-fasting status of an organism.^1^ We recently identified the ureahydrolase, arginase 2 (Arg2), as a fasting-induced hepatocyte factor, the induced expression of which is sufficient to enhance hepatocyte mitochondrial function, block hepatic triglyceride accumulation, and increase peripheral insulin sensitivity.^2^ These findings align with the broader consideration that the arginases represent tractable targets to treat metabolic disease and beyond.

A key limitation to optimally modulate arginase signaling and its related pathways is an incomplete understanding of the physiological effects of hepatocyte arginase activity, and a lack of clarity regarding structural determinants of arginase action.^3^^,^^4^ For example, Arg1 mutations in humans induce a classical urea cycle defect (UCD),^5^^,^^6^ whereas Arg2 mutations causing classical UCD are not reported. In contrast, inhibiting Arg2 blocks development of pancreatic ductal carcinoma and other tumors,^7^, ^8^, ^9^ asthma pathogenesis,^9^ and renal fibrosis.^10^ Germline whole-body Arg2 deficiency also induces spontaneous steatohepatitis and promotes renal cell carcinoma progression,^11^ and activating Arg2 promotes T cell energetics and fitness.^12^ In addition, these apparently paradoxical findings regarding the role of Arg2 in health and disease indicate that Arg2 function depends highly upon cell- and pathophysiological context.

Beyond such considerations, abundant data further demonstrate that Arg2 enzymatic activity is surprisingly dispensable for some of its critical Arg2 functions. This includes ureahydrolysis-independent suppression of endothelial autophagy and atherosclerosis,^13^ cell migration and adhesion,^14^ and vascular smooth muscle senescence and apoptosis.^15^ The data indicate that Arg2 function is complex, physiologically important, and incompletely defined. Moreover, despite high homology, Arg1 and Arg2 retain distinct physiologic functions.

Recently, we demonstrated that hepatocyte-specific Arg2 expression promotes insulin sensitivity and energy expenditure in obese mice. In addition, we showed that systemically administering pegylated arginine deiminase (ADI-PEG20), similarly improved glucose homeostasis and enhanced hepatocyte mitochondrial oxidation^16^, ^17^, ^18^ by inducing hepatocyte autophagic flux and FGF21 secretion.^17^ In contrast with prior data demonstrating broad hydrolysis-independent function for Arg2, our data prompt the assertion that at least part of the metabolic sequelae of hepatocyte arginase activation depends on its ability to regulate cellular arginine. This is especially important, as a primary structural distinction between Arg1 and Arg2 is the Arg2 mitochondrial targeting sequence (MTS), which could define the Arg1 and Arg2 substrate pools. We therefore sought to understand the structure-function relationship governing the metabolic actions of hepatocyte Arg2.

To that end, we leveraged genetically obese and hepatocyte-specific Arg2-deficient (Arg2^LKO^) mice and a genetic complementation approach to elucidate the interrelationship between Arg2 mitochondrial localization, ureahydrolysis, and its effects on hepatocyte and extrahepatic oxidative metabolism. We demonstrate that Arg2 suppresses hepatic lipid accumulation and promotes hepatic and peripheral insulin signaling largely independent of both enzymatic activity and mitochondrial localization. Arg2 similarly enhanced caloric expenditure independent of its hydrolase activity. In contrast, the ability of Arg2 to promote peripheral insulin sensitivity as well as hepatocellular and peripheral glucose oxidation require both intact Arg2 mitochondrial localization and ureahydrolysis. Together, these new data define a structure-function relationship that governs hepatocyte Arg2 metabolic function, identify specific metabolic processes in which mitochondrial arginine may participate, and nominate mitochondrial ureahydrolysis as a key determinant of glucose oxidative capacity in mammals.

## Results

### Histidine 160 and the N-terminal Mitochondrial Targeting Sequence Mediate Arg2 Ureahydrolysis and Subcellular Localization

**Figure 1:**
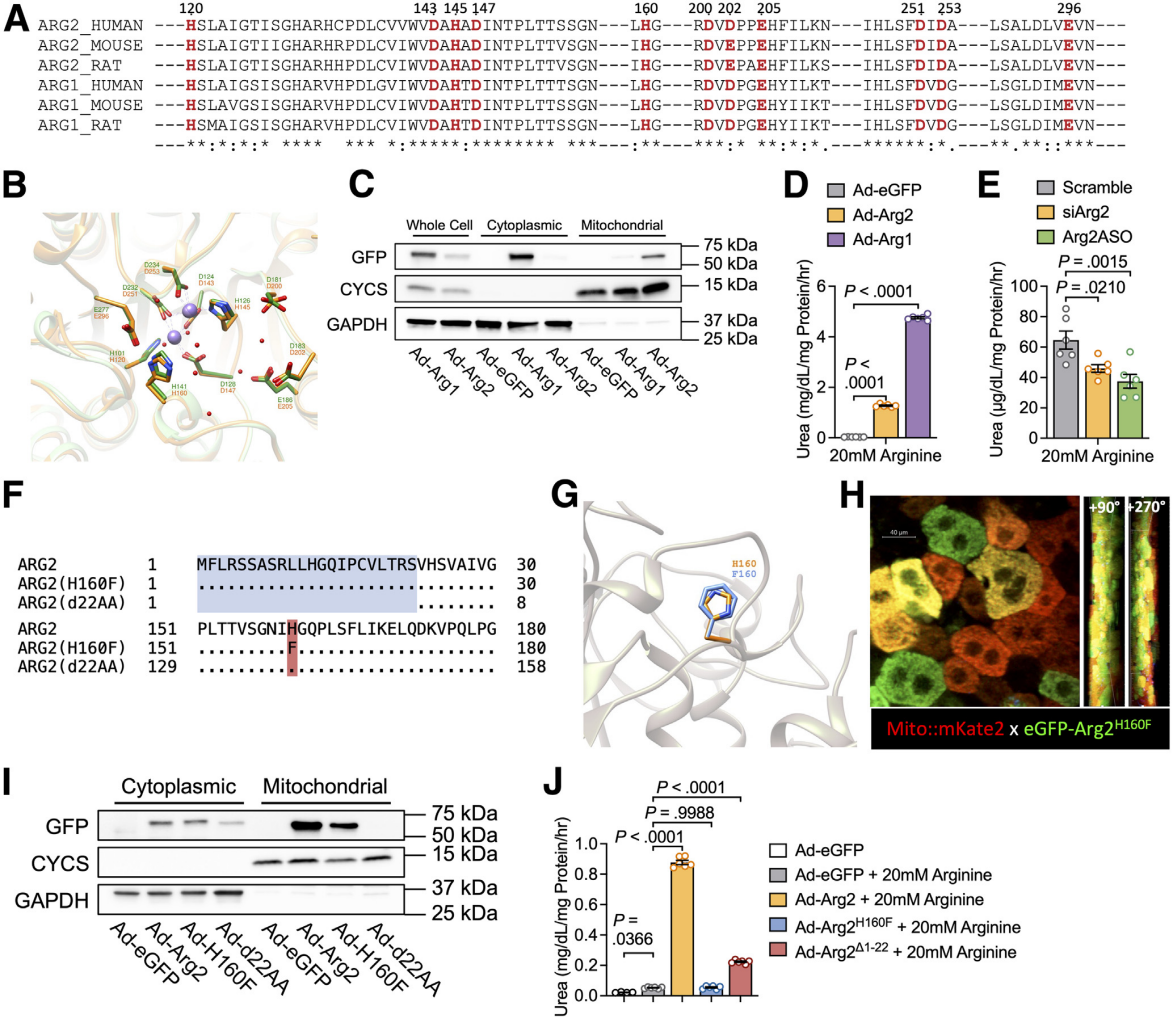
**Arg2 contains conserved functional domains that mediate ureahydrolysis and mitochondrial localization.***A*, Multiple amino acid sequence alignment of both isoforms of arginase from human, rat, and mouse. The conserved active-site residues involved in arginine binding and hydrolysis are highlighted in *red*. *B*, Superposition of human Arg1 (*green*) and Arg2 (*orange*) structure with active sites highlighted in (*A*). Molecular representation was generated using PDB accession codes 2ZAV (ARG1) and 1PQ3 (ARG2) using UCSF Chimera. *C*, Immunoblot analysis of GFP protein levels in fractionated adenovirus-treated AML12 hepatocytes overexpressing eGFP-tagged ARG1 or ARG2 with eGFP alone as the control. GAPDH and CYCS are used as cytoplasmic and mitochondrial loading and fractionation enrichment controls, respectively. *D*, Arginine-stimulated ureagenesis in glucose- and serum-deprived AML12 murine hepatocytes overexpressing eGFP, ARG2, or ARG1 in vitro. (n = 6 independent cultures per group stimulated with arginine, pooled data from 2 independent experiments). *E*, Arginine-stimulated ureagenesis in glucose- and serum-deprived Arg2-deficient AML12 murine hepatocytes. Cultures were treated with siRNA, ASO, or mock transfected 48 hours prior to ureagenesis assay. n = 5–6 per group, pooled from 2 independent experiments. *F*, Sequence alignment of wild-type Arg2 and Arg2 mutants (Arg2^H160F^ and Arg2^Δ1-22^) showing the point mutation at Histidine 160, and the deletion of amino acid residues 1-22 of mouse Arg2. *G*, Superposition of wild-type Arg2 domain architecture with Arg2^H160F^. *H*, Intravital 2-photon imaging depicting the localization of eGFP-tagged Arg2^H160F^ in livers of *mito::mKate2* mice (*mitochondria labeled red*). Scale bar: 40 μm. 90° and 270° X-planar rotations are shown at right. *I*, Immunoblot analysis of GFP protein levels in fractionated adenovirus-treated AML12 hepatocytes overexpressing eGFP-tagged Arg2, Arg2^H160F^, and Arg2^Δ1-22^. eGFP alone localization serves as the control. GAPDH and CYCS/PDHA/HSP60 are demonstrated as cytoplasmic and mitochondrial loading controls, respectively. *J*, Arginine-stimulated ureagenesis in glucose- and serum-deprived AML12 hepatocytes overexpressing eGFP, Arg2, Arg2^H160F^, or Arg2^Δ1-22^ in vitro. n = 4 independent cultures for starvation control, and n = 6 independent cultures per group for arginine-treated groups. Data are represented as mean ± standard error of the mean. Each data point represents an individual animal or an independent culture. Exact *P*-values are shown. Statistical significance was determined by using 1-way analysis of variance with Dunnett’s multiple-comparisons test in *D*, *E*, and *J*.

The mammalian arginine ureahydrolases, Arg1 and Arg2, hydrolyze arginine to ornithine and urea. We reported that fasting induces hepatocyte Arg2 expression and that forced Arg2 expression induces thermogenesis and insulin sensitization in genetic and diet-induced obese animals.^2^ Encoded by different genes, Arg1 and Arg2 nevertheless share approximately 61% amino acid sequence identity.^3^^,^^4^ Multiple sequence alignment analysis revealed cross-mammalian conservation of substrate-binding active-site residues (eg, H160), the binuclear Mn^2+^ cluster core (Figure 1*A* and 1*B*), and the 3-dimensional active site conformation of the each isozyme, by in silico modeling of the Arg1 and Arg2 active sites (Figure 1*B*). We performed subcellular hepatocyte cytosolic and mitochondrial enrichment after overexpressing GFP, GFP-tagged Arg1, or GFP-tagged Arg2. We first confirmed cytoplasmic and mitochondrial-enriched fractions by immunoblotting fractions for sub-cellular mitochondrial and cytoplasmic markers, CYCS and GAPDH, respectively (Figure 1*C*). GFP immunoblot analysis revealed predominant Arg1 expression in the cytosolic fraction, whereas Arg2 localized to the mitochondrial fraction (Figure 1*C*). We compared Arg1 and Arg2 activity by quantifying urea generation in hepatocytes overexpressing GFP, Arg1, or Arg2. Both Arg1 and Arg2 overexpression markedly increased hepatocyte ureahydrolase activity when compared with GFP-expressing hepatocytes, and Arg1 expression increased ureagenesis to a ∼3-fold greater extent vs Arg2-expressing hepatocytes (Figure 1*D*). We then used orthogonal siRNA- and antisense oligonucleotide-based methods to reduce hepatocyte Arg2 expression in cultured hepatocytes. Surprisingly, despite lower specific activity and cellular abundance, Arg2 knockdown significantly impaired basal arginine conversion to urea (Figure 1*E*) by approximately 30% when compared with scrambled nucleotide-treated cultures. As a reference point, inducible Arg1 deletion impaired ureagenesis in liver by 60% to 80%.^19^ Our data thus indicate that Arg1 and Arg2 together mediate the majority of basal hepatic ureagenesis.

To further define the structure-function relationship between Arg2 localization, arginine catabolism, and the metabolic effects of Arg2 expression, we generated 2 Arg2 mutant constructs. First, we deleted amino acid residues 1-22 of Arg2 (Arg2 ^Δ1-22^), corresponding to the putative MTS (Figure 1*F*, blue-highlighted residues). We also generated an Arg2 mutant in which histidine 160 is mutated to a phenylalanine (Arg2^H160F^). This mutation is previously demonstrated to ablate ureahydrolytic activity^13^ (Figure 1*F*, orange-highlighted residue, and Figure 1*G*). We leveraged our mito::*mKate2* mice harboring red fluorescence-labeled mitochondria^20^ to ask if GFP-tagged Arg2^H160F^ localizes to the mitochondria. Prior data indicated sub-total transgenic mKate2 expression in hepatocytes of these mice.^20^ In vivo 2-photon microscopy in mito::*mKate2* mice expressing GFP-Arg2^H160F^ corroborated this finding and further demonstrated Arg2^H160F^ co-localization with mitochondria, as indicated by yellow-spectral fluorescence (Figure 1*H*). We aimed to confirm or refute this finding biochemically in each of our 3 Arg2 constructs through mitochondrial and cytosolic fractionation. This revealed prominent Arg2 and Arg2^H160F^ bands in the enriched mitochondrial fraction, whereas no Arg2 ^Δ1-22^ band was observed in the mitochondrial fraction. This suggested that the first 22 amino acids in Arg2 mediate its mitochondrial localization (Figure 1*I*).

We then quantified ureagenic capacity of Arg2, Arg2^H160F^, and Arg2 ^Δ1-22^ by overexpressing wild-type Arg2, Arg2^H160F^, and Arg2^Δ1-22^ in AML12 hepatocytes prior to incubation in glucose-free media and arginine refeeding (6-hour refeeding period). Wild-type Arg2 and Arg2^Δ1-22^ overexpression increased ureagenesis when compared with GFP- and Arg2^H160F^-expressing hepatocytes (Figure 1*J*). Taken together, Arg2 mediates basal hepatocyte mitochondrial ureagenesis, which justifies further dissection of the structural determinants of its metabolic effects.

### Arg2 Induces Whole-body Thermogenesis in Obese Mice Independent of Ureahydrolysis

**Figure 2:**
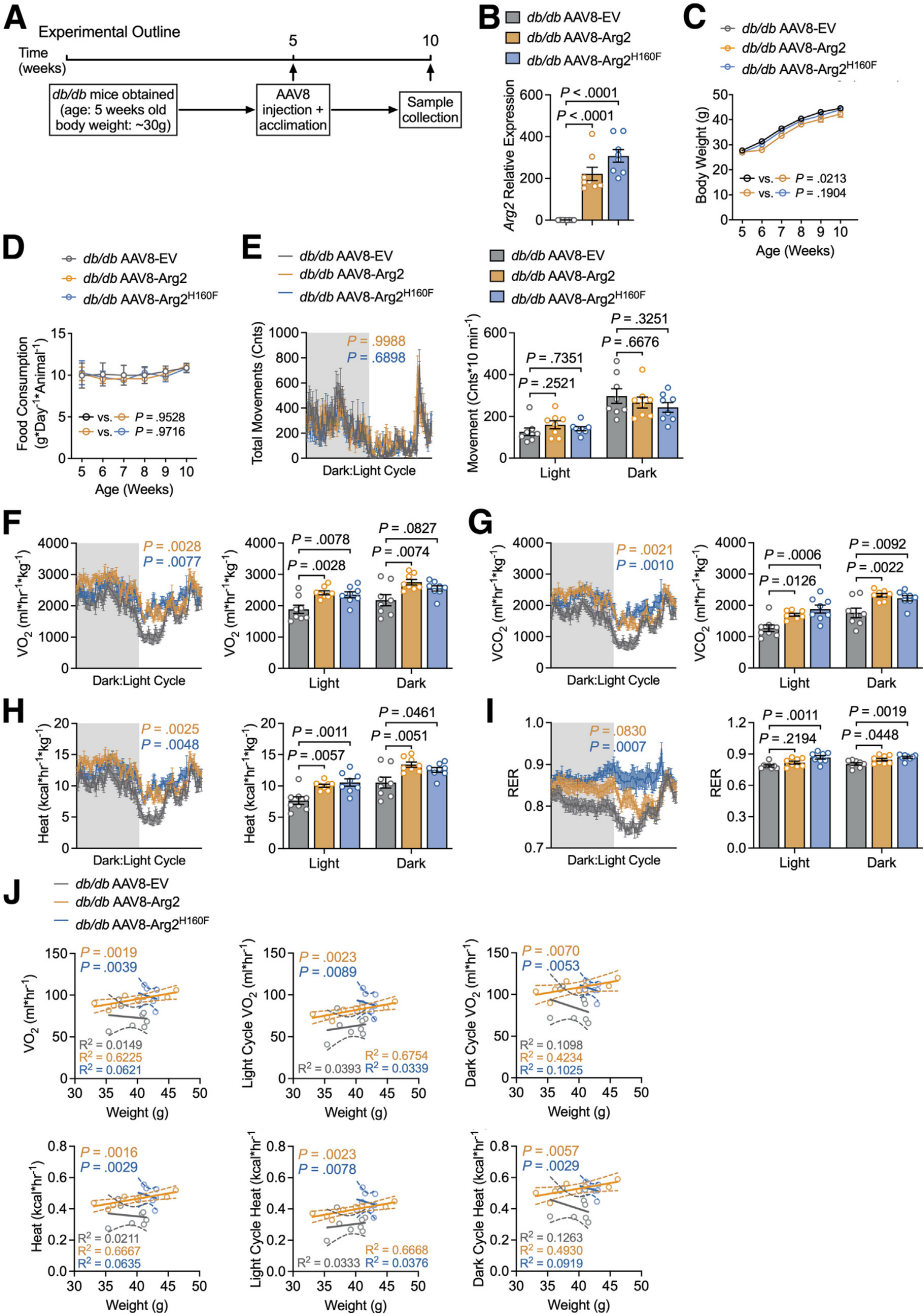
**Arg2 induces whole-body thermogenesis independent of its enzymatic function.***A*, Experimental schematic describing intervention and timing of assays in *db/db* mice. *B*, qRT-PCR quantification of *Arg2* in liver from *db/db* mice treated with control AAV8- EV, AAV8-Arg2, or AAV8-Arg2^H160F^. Gene expression was normalized to *36B4* expression. *C*, Body weight versus time in mice *db/db* mice expressing EV, Arg2, or Arg2^H160F^. n = 8 mice per group. *P*-values represent statistical differences in line slope and intercept. *D*, Daily food consumption measured in *db/db* mice expressing EV, Arg2, or Arg2^H160F^ (n = 8 mice per group). *E*, Indirect calorimetry demonstrating light- and dark-cycle locomotion in *db/db* mice expressing EV, Arg2, or Arg2^H160F^. Means are quantified at right. *P*-values are shown, calculated by 1-way analysis of variance with Dunnett’s multiple-comparisons test. *F– I*, Light- and dark-cycle indirect calorimetry showing measurements of oxygen consumption (VO_2_, *F*), carbon dioxide output (VCO_2_, *G*), energy expenditure (Heat, *H*), and RER (*I*) in *db/db* mice expressing EV, Arg2 or Arg2^H160F^ (n = 8 mice per group), 24-hour tracing is depicted after a 48- to 72-hour acclimatization period) quantified by indirect calorimetry. Quantified VO_2_, VCO_2_, energy expenditure, and RER during the light and dark cycle (n = 8 mice per group) are shown on the right. The dark shaded area indicates the 12-hour dark cycle, and the clear open area indicates the 12-hour light cycle. *J*, Body weight vs energy expenditure regression plots during light and dark cycles of *db/db* mice expressing Arg2 or its mutant constructs in vivo (n = 8 mice per group). Data are represented as mean ± standard error of the mean. Each data point represents an individual animal. Exact *P*-values are shown. Statistical significance was determined using 2-way analysis of variance with Dunnett’s multiple-comparisons test in *C*, *D*, and left panels in *E–I*. Statistical significance was determined using 1-way analysis of variance with Dunnett’s multiple-comparisons test in *B*, and right panels in *E–I*.

Prior data indicate reduced hepatocyte Arg2 expression in over-fed, fructose-exposed murine hepatocytes and in obese (*db/db*) mice.^2^ To define the physiological role of Arg2-mediated hepatocyte arginine catabolism, we administered adeno-associated vector (AAV8) encoding Arg2, mutant Arg2^H160F^, or empty vector (EV) in 5-week-old *db/db* mice. The encoded genes of interest are each driven by the hepatocyte-specific thyroxine-binding globulin (TBG) promoter. Five weeks post-AAV8 delivery, we subjected all mice to a battery of metabolic assays (experimental outline shown in Figure 2*A*). We first performed real-time quantitative reverse transcription polymerase chain reaction (qRT-PCR) in liver to validate Arg2 and Arg2^H160F^ overexpression relative to liver in *db/db* mice expressing control vector (Figure 2*B*)**.** Hepatocyte-specific Arg2 overexpression significantly lowered the total body mass-time interaction (Figure 2*C*), whereas Arg2^H160F^ did not. Neither Arg2 nor Arg2^H160F^ altered food consumption or locomotion over the dark-light cycle (Figure 2*D* and 2*E*).

Additionally, hepatocyte-specific Arg2 and Arg2^H160F^ each increased whole-body oxygen consumption (VO_2_), VCO_2,_ and energy expenditure throughout both dark and light cycles (Figure 2*F–H*). In addition, both Arg2 and Arg2^H160F^ increased dark cycle respiratory exchange ratio (RER) when compared with vector-treated *db/db* controls, whereas Arg2^H160F^ also increased light cycle RER (Figure 2*I*). Regression analyses revealed these increases remain apparent when also controlling for significant body weight change in Arg2-overexpressing mice by analysis of covariance (Figure 2*J*). Hepatocyte Arg2 thus induced heat generation and glucose oxidative predilection independent of its ureahydrolytic activity. Overall, we observed modestly increased heat generation in Arg2- and Arg2^H160F^-overexpressing mice without body weight changes across groups. Specifically, we observed daily food intake of ∼45 kcal/mouse/day across groups (Figure 2*D*), whereas heat generation was 6.7 to 10.6 kcal/mouse/day (Figure 2*H*). This represents a 5- to 7-fold excess caloric intake over energy expenditure. We thus cannot rule out that large excesses in caloric intake may obscure any weight changes that might occur due to smaller-magnitude improvements in energy expenditure in Arg2- and Arg2^H160F^-overexpressing mice.

### Arg2 Ureahydrolytic Activity Mediates Arg2 Protection From Proinflammatory Cytokine Expression, but not Hepatic Steatosis, in Genetically Obese Mice

**Figure 3:**
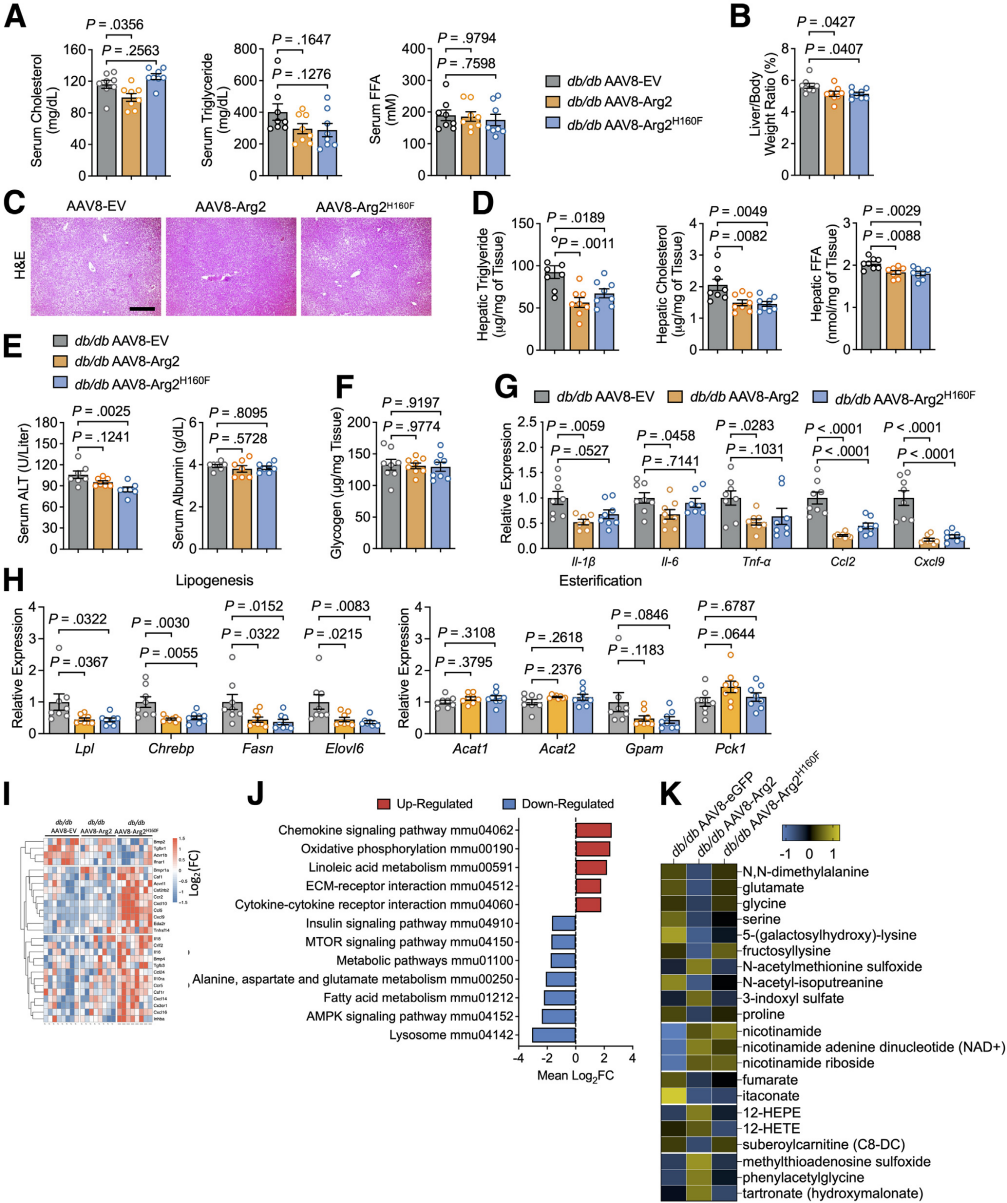
**Enzyme-dead ARG2**^**H160F**^**improves liver steatosis but promotes proinflammatory gene expression.***A*, Enzymatic-colorimetric based quantification of serum triglyceride, cholesterol, and non-esterified fatty acid in *db/db* mice expressing EV, Arg2 or Arg2^H160F^(n = 8 mice per group). *B*, Liver weight-to-body weight ratio in *db/db* mice expressing EV, Arg2, or Arg2^H160F^. *C*, Representative liver sections from *db/db* mice expressing EV, Arg2, or Arg2^H160F^ stained with H&E. Scale bar, 100 μm. *D*, Quantification of hepatic triglyceride, cholesterol, and non-esterified fatty acid content in chloroform: methanol lipid extracts in livers from *db/db* mice expressing EV, Arg2, or Arg2^H160F^ (n = 8 mice per group). *E*, Enzymatic activity of ALT (*left*) and albumin (*right*) concentration in *db/db* mice expressing EV, Arg2, or Arg2^H160F^ (n = 8 mice per group). *F*, Quantification of hepatic glycogen content in livers from *db/db* mice expressing hepatocyte-specific EV, Arg2, or Arg2^H160F^ (n = 8 mice per group). *G*, RT-qPCR analysis of proinflammatory gene expression in livers from *db/db* mice expressing hepatocyte-specific EV, Arg2, or Arg2^H160F^ (n = 8 mice per group). Gene expression was normalized to *36B4* expression. *H*, RT-qPCR analysis of lipogenesis- and esterification-related gene expressions in livers from *db/db* mice expressing hepatocyte-specific EV, Arg2, or Arg2^H160F^ (n = 8 mice per group). Gene expression was normalized to *36B4* expression. *I*, Unsupervised clustering of differentially expressed genes as quantified by bulk transcriptomics in livers from *db/db* mice expressing EV, Arg2, or Arg2^H160F^. *J*, KEGG pathway enrichment analysis demonstrating up- and down-regulated signaling and metabolism pathways based on differentially expressed genes in Arg2^H160F^ vs Arg2-expressing *db/db* mouse liver. *K*, Quantitative metabolomics, as quantified by mass spectrometric analysis in livers from *db/db* mice expressing EV, Arg2, or Arg2^H160F^. Mapped are metabolite groups that are statistically different by 1-way analysis of variance. Data are represented as mean ± standard error of the mean. Each data point represents an individual animal or an independent culture. Exact *P*-values are shown. Statistical significance was determined using 1-way analysis of variance with Dunnett’s multiple-comparisons test in *A*, *B*, *D*, *E*, *F*, *G*, and *H*.

We next quantified the contribution of Arg2-mediated ureahydrolysis to hepatic and plasma lipid accumulation and hepatic inflammatory gene expression in obese, diabetic mice. Hepatocyte-specific Arg2 overexpression in *db/db* mice lowered plasma cholesterol when compared with AAV8 vector controls, whereas Arg2^H160F^ did not (Figure 3*A*). Arg2 and Arg^H160F^ both produced lower-trending plasma triglycerides (TGs), which did not reach statistical significance when compared with control vector-expressing *db/db* mice.

Gross, microscopic, and biochemical analysis of the liver, however, first revealed lower liver weight-to-body weight ratio (Figure 3*B*), lower histologic parenchymal vacuolization (Figure 3*C*), and lower intrahepatic TGs, cholesterol, and non-esterified fatty acid accumulation in *db/db* mice expressing either Arg2 or Arg2^H160F^ (Figure 3*D*) vs vector control-treated *db/db* mice. Serum alanine aminotransferase (ALT) in both *db/db* Arg2 and *db/db* Arg2^H160F^ trended lower than in *db/db* vector controls, and this reached significance in *db/db* Arg2^H160F^ mice, without changes in serum albumin or intrahepatic glycogen in any group relative to control *db/db* mice (Figure 3*E* and 3*F*).

qRT-PCR analysis also revealed decreased proinflammatory cytokine (eg, IL1 β, IL6, TNFα) and chemokine (eg, CCL2, CXCL9) gene expression in liver of *db/db* Arg2 mice. This anti-inflammatory effect was blunted in *db/db* Arg2^H160F^ mice, wherein *db/db* Arg2^H160F^ significantly suppressed only chemokine expression (Figure 3*G*). In contrast, both Arg2 and Arg2^H160F^ overexpression blunted hepatic lipogenic gene expression in *db/db* mice without altering gene expression in the lipid esterification pathway (Figure 3*H*).

We sought to agnostically refute or corroborate this differential effect on hepatic inflammation. To that end, we performed bulk transcriptomic analysis of livers derived from *db/db* Arg2, and Arg2^H160F^ mice. Unsupervised clustering revealed differential gene expression in several clusters of hepatic genes when comparing either Arg2-expressing mutant liver with vector controls (Figure 3*I*). Kyoto Encyclopedia of Genes and Genomes (KEGG) pathway enrichment analysis of these differentially expressed genes revealed significantly increased cytokine-cytokine receptor interaction and chemokine signaling pathway gene expression in *db/db* Arg2^H160F^ relative to *db/db* Arg2 mice. Conversely, we observed significant downregulation of mTOR and insulin signaling pathway gene expression in *db/db* Arg2^H160F^ relative to *db/db* Arg2 mice (Figure 3*J*). Associated with improvements in liver and peripheral lipid accumulation and improvements in chemokine signaling in both *db/db* Arg2 and Arg2^H160F^ mice, shotgun metabolomic analysis of liver in each group revealed significantly increased nicotinamide adenine dinucleotide (NAD^+^) pathway intermediaries: nicotinamide, NAD^+^ and nicotinamide riboside (NR) in both *db/db* Arg2 and Arg2^H160F^ mice when compared with vector control mice (Figure 3*K*). The data together indicate that Arg2 exerts its NAD^+^, anti-steatotic, and part of its anti-inflammatory effects independent of ureahydrolytic activity.

### Enzymatic Activity is Required for Optimal Insulin Sensitization Downstream of Hepatocyte Arg2 Expression in Obese Mice

**Figure 4:**
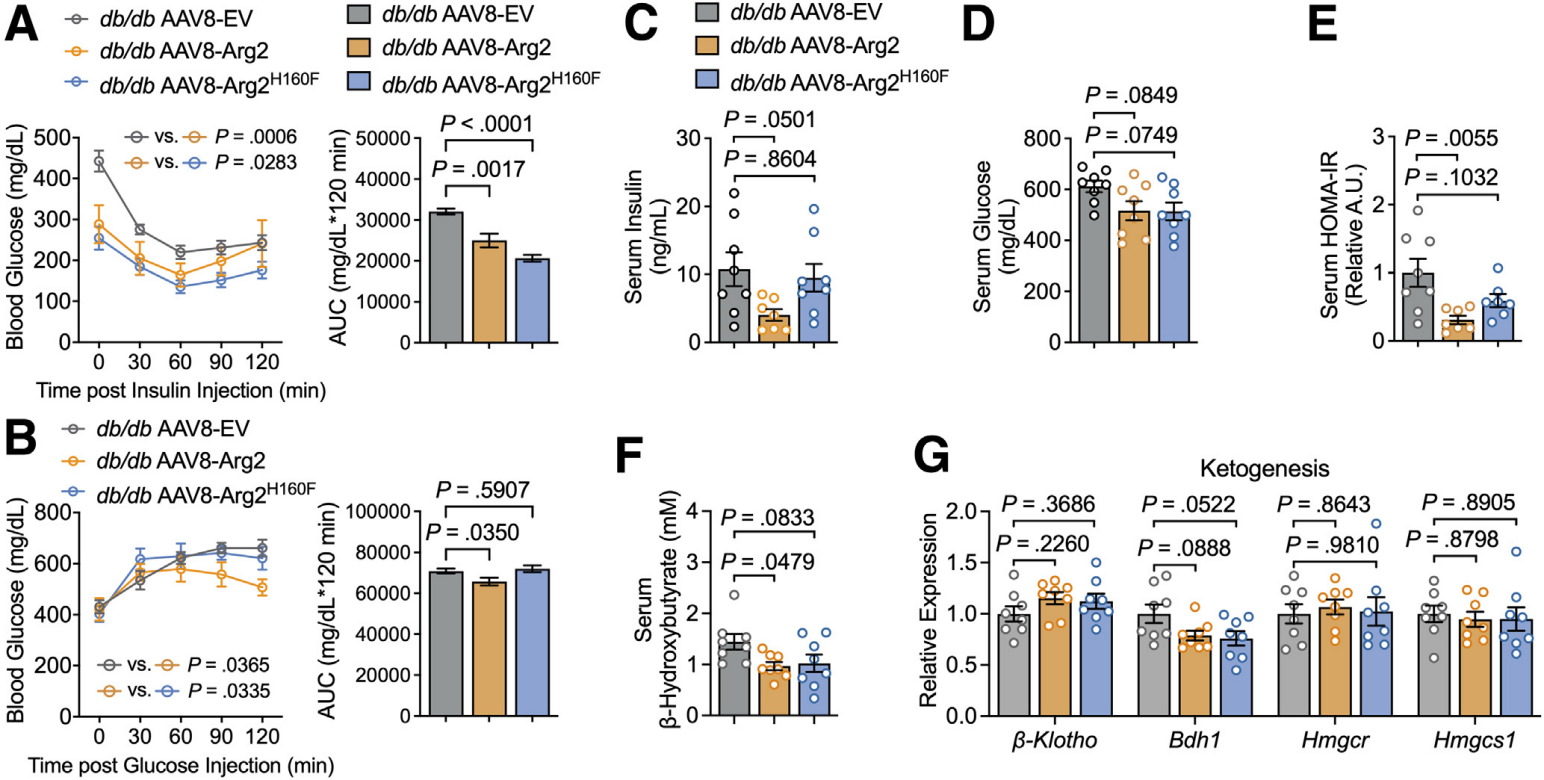
**Arg2 exerts hydrolysis-dependent imp peripheral glucose homeostasis.***A and B*, Intraperitoneal GTTs (*A*) and ITTs (*B*) from *db/db* mice expressing EV, Arg2 or Arg2^H160F^ (n = 8 mice per group). Glucose-time curves are shown on left, and total glucose-time AUC is shown at right in each panel. *C–F*, Random-fed serum glucose (*C*), serum insulin (*D*), calculated HOMA-IR (*E*), and serum β-hydroxybutyrate (*F*) in AAV8-treated *db/db* mice (n = 8 mice per group). *G*, RT-qPCR analysis of ketogenesis-related gene expression in livers from *db/db* mice expressing hepatocyte-specific EV, Arg2 or Arg2^H160F^ (n = 8 mice per group). Gene expression was normalized to *36B4* expression. Data are represented as mean ± standard error of the mean. Each data point represents an individual animal. Exact *P*-values are shown. Statistical significance was determined using 2-way analysis of variance with Dunnett’s multiple-comparisons test in left panels in *A* and *B*. Statistical significance was determined using 1-way analysis of variance with Dunnett’s multiple-comparisons test in right panels in *A* and *B*, *C*, *D*, *E*, *F*, and *G*.

Arg2 exerts important physiological function (eg, increased heat generation, blockade of hepatic steatosis) independent of its enzymatic competency. We extended this analysis to define the extent to which ureahydrolysis mediates the peripheral glucose homeostatic effect of Arg2. *db/db* mice expressing EV, Arg2, or Arg2^H160F^ were subjected to insulin tolerance testing (ITT). This revealed significantly lower glucose-time curves in *db/db* Arg2 and Arg2^H160F^ mice when compared with vector control mice. (Figure 4*A*). However, *db/db* Arg2 had significantly lower glucose tolerance testing (GTT) areas under the curve (AUC), fasting insulin, and lower homeostatic model assessment for insulin resistance (HOMA-IR) vs *db/db* controls. Yet, we observed no significant improvements in *db/db* Arg2^H160F^ GTT AUC (Figure 4*B*), HOMA-IR, or serum insulin when compared with the *db/db* controls (Figure 4*C–E*). Finally, we quantified serum ketones and hepatic ketogenic gene expression in mice expressing control vector, Arg2, or Arg2^H160F^. Hepatocyte Arg2 expression reduced serum β-hydroxybutyrate (Figure 4*F*), without altering hepatic ketogenic gene expression (Figure 4*G*). In contrast, neither serum ketones nor hepatic ketogenic gene expression was altered in mice overexpressing Arg2^H160F^ (Figure 4*F–G*). The data are consistent with our insulin resistance data (Figure 4*E*). Specifically, Arg2 improves HOMA-IR in *db/db* mice, but Arg2^H160F^ does not. Because peripheral insulin suppresses serum ketosis, it follows that hepatocyte Arg2 overexpression suppresses serum ketone accumulation, when compared with control mice.

### Mitochondrial Localization is Dispensable for the Anti-steatotic, but not Insulin-sensitizing Effects of Hepatocyte Arg2

**Figure 5:**
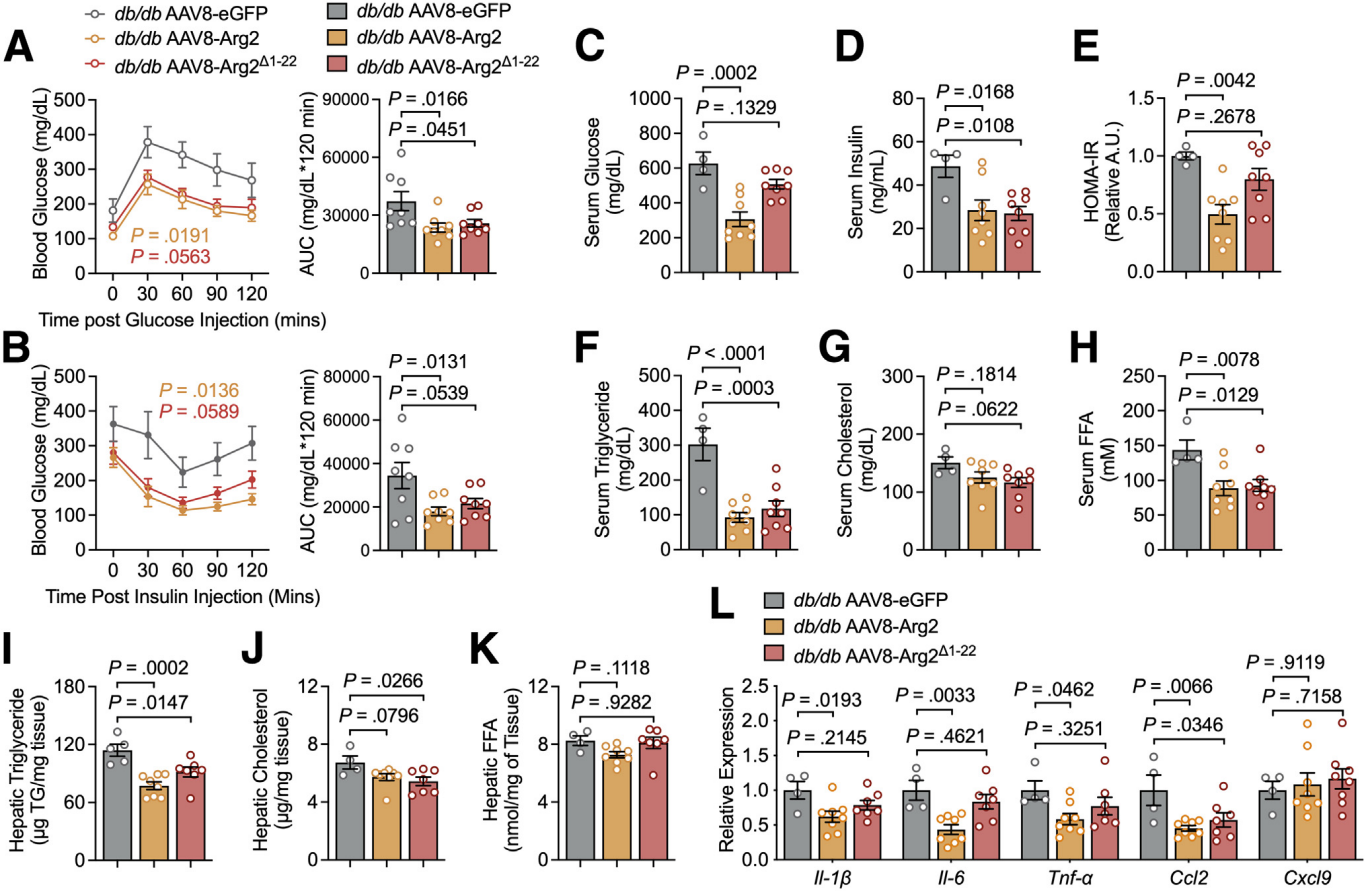
**Arg2 mitochondrial localization is required for improvements in insulin sensitivity.***A and B*, Intraperitoneal GTTs (*A*) and ITTs (*B*) in AA *db/db* mice expressing GFP, Arg2, or Arg2^Δ1-22^ (n = 8 mice per group, respectively). Glucose-time curves are shown at left, and total glucose-time AUC is shown at right in each panel. *C–E*, Serum glucose (*C*), serum insulin (*D*), and HOMA-IR (*E*) in ad libitum-fed *db/db* mice expressing GFP, Arg2, or Arg2^Δ1-22^ (n = 8 mice per group). *F–H*, Serum TG (*F*), cholesterol (*G*), and non-esterified fatty acids (*H*) in ad libitum-fed *db/db* mice expressing GFP, Arg2, or Arg2^Δ1-22^. *I–K*, Hepatic TG (*I*), cholesterol (*J*), and non-esterified fatty acids (*K*) in chloroform:methanol extracts from livers of ad libitum-fed *db/db* mice expressing GFP, Arg2, or Arg2^Δ1-22^. *L*, RT-qPCR analysis of proinflammatory gene expression in livers from *db/db* mice expressing hepatocyte-specific EV, Arg2 or Arg2^H160F^ (n = 8 mice per group). Gene expression was normalized to *36B4* expression. Data are represented as mean ± standard error of the mean. Each data point represents an individual animal or an independent culture. Exact *P*-values are shown. Statistical significance was determined using 2-way analysis of variance with Bonferroni’s multiple-comparisons test in *A* and *B*. Statistical significance was determined using 1-way analysis of variance with Dunnett’s multiple-comparisons test in *C–L*.

Deletion of amino acids 1-22 of Arg2 abrogates its ability to localize to the mitochondria while retaining its arginine hydrolysis (Figure 1). We expressed Arg2, EV, or Arg2^Δ1-22^ in *db/db* mice to test how mistargeting Arg2 away from the mitochondrion modulates the metabolic effects of Arg2. Five weeks after GFP, Arg2, or Arg2^Δ1-22^ induction, GTT and ITT revealed improved ITT and GTT curves and AUC in Arg2-overxpressing *db/db* mice. However, GTT AUC and time-glucose curves were improved in *db/db* Arg2 and Arg2^Δ1-22^ mice, but not the ITT AUC (Figure 5*A–B*). In addition, Arg2 and Arg2^H160F^ expression decreased serum insulin, but Arg2 suppressive effects on serum glucose and overall HOMA-IR were reversed in *db/db* Arg2^H160F^ mice (Figure 5*C–E*).

Analysis of peripheral and hepatic lipid accumulation yielded significant reductions in serum TGs and nonesterified fatty acids in both *db/db* AAV8-Arg2 mice and *db/db* Arg2^Δ1-22^ mice without significant reduction in serum cholesterol in either group when compared with GFP-expressing mice (Figure 5*F–H*). Both Arg2 and Arg2^Δ1-22^ had significantly lower hepatic TGs. Only Arg2, but not Arg2^Δ1-22^, lowered hepatic cholesterol (Figure 5*I–K*). Furthermore, the Arg2-mediated reduction in pro-inflammatory cytokine gene expression was largely abolished in *db/db* Arg2^Δ1-22^ mice. The exception to this was in CCL2 gene expression, which was suppressed both by Arg2 and Arg2^Δ1-22^ (Figure 5*L*). The MTS of Arg2 is thus required for full glycemic control but is dispensable for Arg2 to block hepatic steatosis.

### Hepatocyte Arg2 Promotes Hepatic and Peripheral Insulin Sensitivity and Suppresses Hepatic Glucose Output

**Figure 6:**
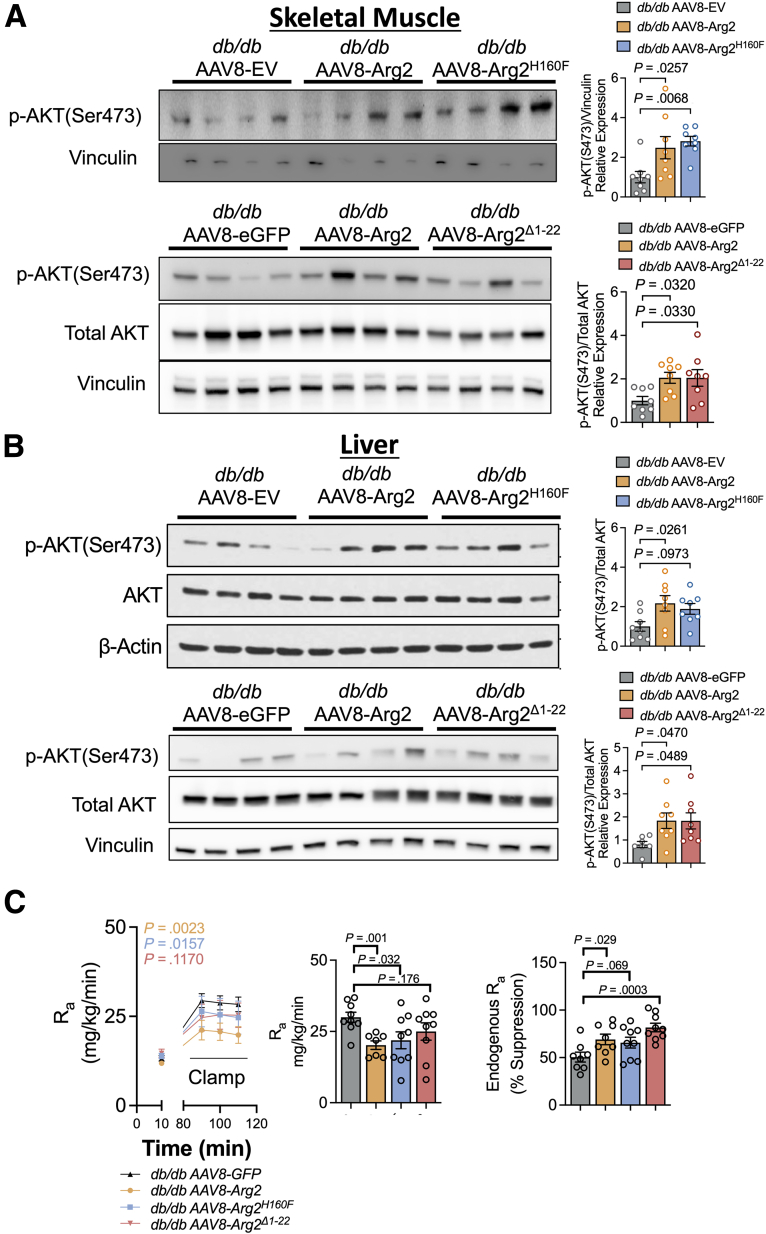
**Hydrolysis- and localization-independent insulin sensitization by ARG2.***A*, Immunoblot analysis of phosphorylated AKT in skeletal muscle from *db/db* mice expressing control empty vector, Arg2, Arg2^H160F^ (*upper panels*), or Arg2^Δ1-22^ (*lower panels*) (n = 4 mice per group). Vinculin was used as a loading control. Normalized densitometric quantification of pAKT(S473) is shown at right (n = 8 mice per group). *B*, Immunoblot analysis of phosphorylated AKT in liver from *db/db* mice expressing control vector, Arg2, Arg2^H160F^ (*upper panels*) or Arg2^Δ1-22^ (*lower panels*) (n = 4 mice per group). Total AKT and vinculin were used as a loading control. Normalized densitometric quantification of pAKT(S473) is shown at right (n = 8 mice per group). *C*, Rate of appearance (R_a_) of peripheral glucose in *db/db* mice expressing GFP, Arg2, Arg2^H160F^, or Arg2^Δ1-22^ during euglycemic clamping. R_a_ vs time tracing is shown at left. Mean steady-state R_a_ is quantified at right. n = 8–9 per group. Data are represented as mean ± standard error of the mean. One-way analysis of variance with Dunnett’s multiple-comparisons test was used in *A*, *B*, and *C* (*right*). Two-way analysis of varaiance with Dunnett’s multiple-comparisons test was used in *C* (*left*).

Both enzymatic and mistargeted Arg2^H160F^ and Arg2^Δ1-22^ mutations impaired Arg2-mediated insulin sensitization in *db/db* mice. Glucose homeostasis is broadly a net balance between hepatic glucose output and glucose utilization in liver and the periphery. We therefore ascertained if Arg2, Arg2^H160F^, and Arg2^Δ1-22^ induce hepatic and peripheral AKT phosphorylation as a marker of tissue insulin responsiveness in *ad libitum*-fed *db/db* mouse tissue. Hepatocyte Arg2 and Arg2^Δ1-22^ overexpression in *db/db* mice increased skeletal muscle and hepatic Akt phosphorylation at serine 473 (Figure 6*A–B*). Arg2^H160F^ induced skeletal muscle but not hepatic AKT phosphorylation (Figure 6*A–B*). Counterbalancing this, we assessed hepatic glucose output during a euglycemic clamp and showed improved steady-state and mean R_a_ (Figure 6*C*) in *db/db* Arg2 and Arg2^H160F^ mice but not in *db/db* Arg2^Δ1-22^ mice.

**Figure 7:**
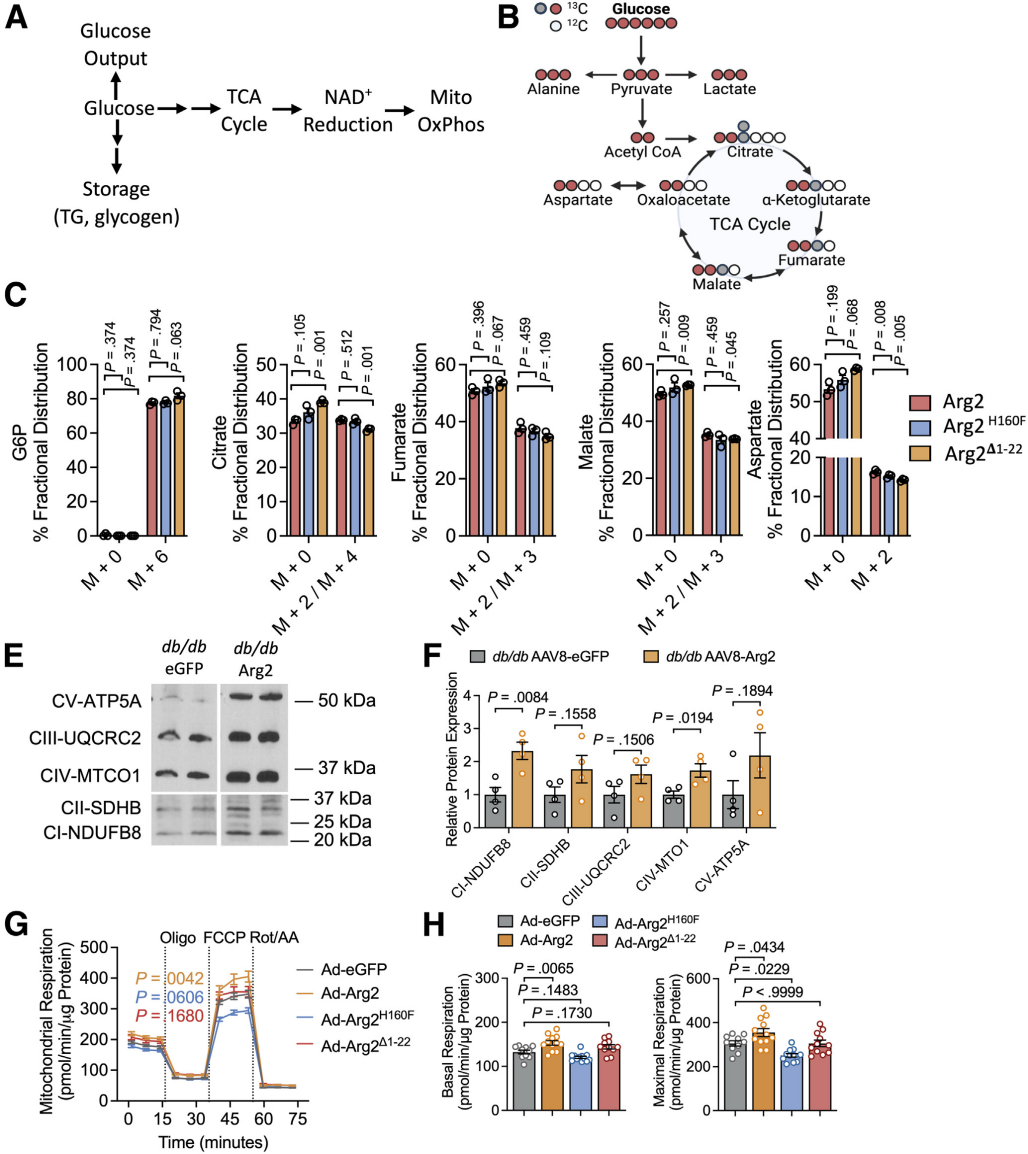
**Ureahydrolysis and mitochondrial localization of ARG2 is required for its effects on glucose oxidation.***A*, Generalized schematic depicting some fates of glucose in the hepatocyte. Glucose is exported, stored, or metabolized in part through the TCA cycle, which produces NADH to drive oxidative phosphorylation. *B*, Stable isotope diagram demonstrating first- (*red*) and second-turn (*gray*) substrate labeling through the TCA cycle. *C*, Fractional distribution of [U-^13^C_6_] in glucose-6-phosphate, TCA cycle intermediaries (citrate, fumarate, malate), and aspartate from universally labeled [U-^13^C_6_] glucose. *E and F*, Immunoblot autoradiograph (*E*) and quantification (*F*) of OXPHOS complexes, including CI subunit NDUFB8, CII, CIII-core protein 2, CIV subunit I, and CV alpha subunit, in livers of *db/db* mice expressing hepatocyte-specific GFP or Arg2. n = 4 per group. *G and H*, Seahorse respirometry showing oxygen consumption vs time (*G*) and quantification of basal and maximal respiration (*H*) in AML12 hepatocytes expressing GFP, Arg2, Arg2^H160F^ or Arg2^Δ1-22^. Data are represented as mean ± standard error of the mean. Each data point represents an individual animal or an independent culture. Exact *P*-values are shown. Statistical significance was determined using 1-way analysis of variance with Dunnett’s multiple-comparisons test in *C* and *H*, 2-way analysis of variance with Dunnett’s multiple-comparisons test in *G*, and Student’s *t*-test was used in *F*.

The data exposed differences in hepatic AKT phosphorylation and glucose output control that prompted us to assess more deeply Arg2 effects on hepatic glucose metabolism and oxidative function. We hypothesized that impaired suppression of glucose output in the setting of mistargeted Arg2^Δ1-22^ would associate with impaired TCA cycle and mitochondrial glucose oxidation in ex vivo hepatocytes from *db/db* Arg2^Δ1-22^ mice (Figure 7*A*).

To define the fate of glucose through the TCA cycle in hepatocytes expressing Arg2, Arg2^H160F^, or Arg2 ^Δ1-22^ we performed stable isotope-labeled glucose tracing through at least 2 rounds of the TCA cycle (Figure 7*B*, red and grey-filled carbon diagrams, respectively). Fractional distribution of unlabeled and glucose-6-phosphate (M + 6) was equivalent across Arg2, Arg2^H160F^, and Arg2^Δ1-22^ groups. This indicated similar steady-state glucose accumulation within the hepatocyte and was consistent with equivalent hepatic insulin signaling responses in each group (Figure 7*B*). However, when compared with Arg2-expressing hepatocytes, Arg2^Δ1-22^-expressing hepatocytes exhibited lower TCA cycle intermediary fractional labeling of citrate (M + 2) and (M + 4) (eg, 2-turn labeling within the TCA cycle), fumarate (M + 2) and (M + 3), and malate (M + 2) and (M + 3) as well as aspartate (M + 2) (Figure 7*C*). The data indicate that Arg2^Δ1-22^ impairs TCA cycle glucose oxidation. Because the TCA cycle reduces NAD^+^ to NADH to provide electron carriers to the mitochondrion, we then measured mitochondrial oxidative capacity by seahorse respirometry, and mitochondrial complex protein abundance in *db/db* Arg2, Arg2^H160F^, and Arg2^Δ1-22^ mice. This revealed that Arg2 induced mitochondrial oxidation, and the abundance of multiple mitochondrial complex proteins–ATP5A, UQCRC2, MTCO1, SDHB and NDUFB8, which was statistically different specifically for complex I and Complex IV proteins (Figure 7*D–E*). Arg2 increased basal and maximal respiration, whereas neither of Arg2 Arg2^H160F^ nor Arg2^Δ1-22^ increased mitochondrial respiration (Figure 7*F–G*).

**Figure 8:**
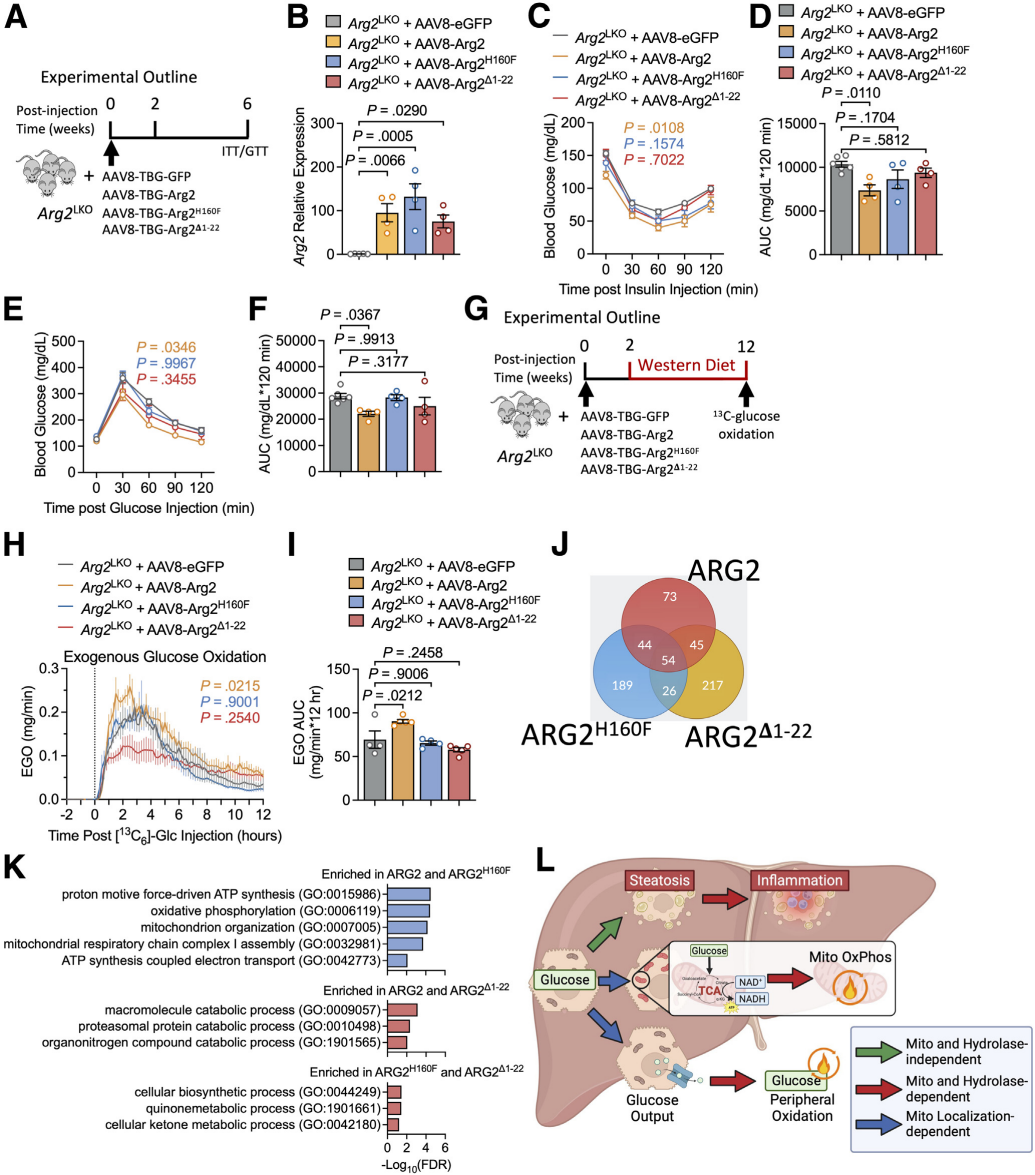
**Genetic complementation reveals ureahydrolysis and mitochondrial localization of ARG2 are required for its effects on glucose oxidation.***A*, Experimental diagram for genetic complementation in hepatocyte-specific Arg2-deficient (*Arg2*^LKO^) mice expressing GFP, Arg2, Arg2, Arg2^H160F^, or Arg2^Δ1-22^. *B*, qRT-PCR quantification of *Arg2* in liver from *Arg2*^LKO^ mice treated with control AAV8, AAV8-Arg2, AAV8-Arg2^H160F^, or AAV8-Arg2^Δ1-22^. *C–F*, Intraperitoneal ITTs (*C and D*) and GTTs (*E and F*) from *Arg2*^LKO^ mice expressing GFP, Arg2, Arg2^H160F^, or Arg2^Δ1-22^ (n = 8 mice per group). Glucose-time curves are shown in *B* and *D*. Glucose-time AUC is shown in *D* and *F*. *G*, Experimental diagram for genetic complementation in hepatocyte-specific Arg2-deficient (*Arg2*^LKO^) mice fed 12-week Western diet expressing GFP, Arg2, Arg2, Arg2^H160F^, or Arg2^Δ1-22^ prior to in vivo glucose oxidation assay. *H and I*, Exogenous glucose oxidation in Western diet-fed *Arg2*^LKO^ mice treated with AAV8 to express either GFP, Arg2, Arg2^H160F^, or Arg2^Δ1-22^ (n = 4, 5, 4, and 5 respectively). [^13^C]-CO_2_ vs. time curve is shown in *G*. Total AUC is shown in *H*. *J*, Venn diagram showing co-immunoprecipitated proteins identified by label-free mass spectrometry in isolated primary hepatocytes treated with adenovirus to overexpress GFP, or GFP-tagged Arg2, Arg2^H160F^, or Arg2^Δ1-22^. The proteins were defined as positive interacting if either (1) absent in the GFP control experiments, or (2) the absolute peptide abundance was greater than 2-fold the amount observed in GFP controls. *K*, GO analysis of the shared proteins between different treatment groups as shown. *L*, Summary of hydrolysis and localization-dependent ARG2 effects on hepatic oxidative metabolism. Data are represented as mean ± standard error of the mean. Each data point represents an individual animal or an independent culture. Exact *P*-values are shown. Statistical significance was determined using 1-way analysis of variance with Dunnett’s multiple-comparisons test in *B*, *D*, *G*, and *I*. Statistical significance was determined using 2-way analysis of variance with Dunnett’s multiple-comparisons test in *C*, *E*, and *H*.

Arg2 expression in fed mice is low, in comparison to Arg1, and yet data here indicate it shares function with Arg1 to mediate basal ureagenesis. We therefore utilized our liver-specific Arg2-deficient (Arg2^LKO^) model, generated by breeding mice with homozygous LoxP insertions surrounding Arg2 Exon 2 with mice expressing Cre recombinase under control of the hepatocyte-specific albumin promoter. We expressed GFP, Arg2, Arg2^H160F^, or Arg2^Δ1-22^ on an Arg2^LKO^ background, and performed insulin and glucose intolerance testing after 4 to 5 weeks (Figure 8*A*). We performed qRT-PCR in liver to validate Arg2 and Arg2 mutant construct overexpression relative to GFP-expressing Arg2^LKO^ liver (Figure 8*B*). ITT and GTT glucose-time curves (Figure 8*C and E*) and AUC (Figure 8*D and F*) were improved in Arg2^LKO^ mice expressing Arg2, but not in Arg2^LKO^ mice expressing Arg2^H160F^ or Arg2^Δ1-22^.

Finally, we assessed whole-body glucose oxidation using heavy isotope, universally-labeled [^13^C]-glucose in vivo. We again utilized a genetic complementation strategy in Western diet-fed Arg2^LKO^ mice overexpressing hepatocyte-specific Arg2, Arg2^H160F^, or Arg2^Δ1-22^, followed by [^13^C]-labeled CO_2_ measurement via indirect calorimetry (Figure 8*G*). Hepatocyte Arg2 complementation in Arg2^LKO^ mice induced whole-body glucose oxidation in vivo, but reconstitution of neither Arg2^Δ1-22^, nor Arg2^H160F^ did the same in the obese state (Figure 8*H and I*).

Finally, we aimed to identify interacting binding partners of Arg2 and its mutants using unbiased mass spectrometry in isolated primary hepatocytes. We treated isolated primary hepatocytes using adenovirus carrying empty vector, GFP, GFP-tagged Arg2, GFP-Arg2^H160F^, or GFP- Arg2^Δ1-22^. We then performed quantitative label-free mass spectrometry on GFP immunoprecipitated complexes to identify potential Arg2 binding partners. Notably, among the shared immunoprecipitated proteins between ARG2 and ARG2(H160F) were proteins involved in mitochondrial function by Gene Ontology (GO) pathway analysis (Figure 8*J–K*). In contrast, consistent with retaining arginine hydrolytic function, proteins that co-immunoprecipitated with Arg2 and GFP- Arg2^Δ1-22^ were enriched in catabolic pathways (Figure 8*K*).

## Discussion

Hepatocyte Arg2 is a fasting-inducible factor that increases insulin sensitivity and hepatic oxidation.^2^ This is phenocopied by pharmacologic, systemic iminohydrolysis of circulating arginine by ADI-PEG20 through FGF21- and autophagic flux-dependent mechanisms.^16^^,^^17^ The data together indicate that Arg2 products ornithine and urea are not fully necessary for its metabolic actions, and that hydrolysis of arginine pools *per se* may drive metabolic effects. However, the critical knowledge gap to optimally target arginase metabolism is in the structural determinants of its control over oxidative metabolism and insulin sensitivity. Here, our data reveal that Arg2 mitochondrial localization and arginine catabolism can partly determine hepatocyte and peripheral oxidative capacity (Figure 8*L*).

Post-prandial insulin exerts 2 major actions to control plasma glucose. First, insulin action in liver suppresses hepatocyte glucose output by blocking processes that provide carbon for glucose production, including glycogenolysis, and autophagic flux. Second, peripheral insulin action induces AKT phosphorylation and glucose uptake from plasma primarily into skeletal muscle depots, whereupon glucose is oxidized and released as CO_2_.^21^ We show here that hepatocyte Arg2 expression improves systemic glycemic control, and this requires both Arg2 ureahydrolytic activity (Figure 4*E*) and mitochondrial localization (Figure 5*E*). We interrogated if this control was due to changes in hepatic glucose output, peripheral glucose oxidation, or both. Mistargeting Arg2 abrogated Arg2 effects on glucose output and TCA cycle flux. In contrast, Arg2 enhanced hepatic glucose oxidation, requiring both ureahydrolytic activity and mitochondrial localization. We conclude that mitochondrial ureahydrolase activity improves glucose homeostasis primarily through augmenting peripheral glucose oxidation. This identifies as a tractable new pathway through which to modulate glucose oxidation in obesity and diabetes.

The data hold implications beyond molecular therapeutics to treat diabetes and obesity, however. They imply a critical distinction between arginine pools in the cell, which are not shared between the arginases. The evidence for this conclusion is as follows. First, the binding partner profile differs greatly when comparing Arg2 and Arg2^H160F^ vs Arg2^Δ1-22^. Both Arg2 and Arg2^H160F^ appear to share oxidative phosphorylation protein scaffolding properties within the mitochondrion (Figure 8*I–J*). Yet, neither Arg2^H160F^ nor Arg2^Δ1-22^ induces mitochondrial oxidative phosphorylation, which diminishes the importance of Arg2-mediated protein scaffolding in the mitochondrion. In contrast, Arg2^Δ1-22^ -expressing hepatocytes exhibit lower TCA cycle flux vs Arg2-expressing hepatocytes. This suggests that even augmenting cytosolic ureahydrolysis is insufficient to drive the TCA cycle as native Arg2. Together the data support a pro-oxidant role either for mitochondrial arginine pool-specific reduction, mitochondrial ornithine generation, or both. In any case, the data strongly support that the mitochondrial pool is compartmentalized from the actions of cytosolic Arg1. Notably, this compartmentalization may also explain why Arg2 fails to compensate for Arg1 mutations clinically in the setting of classical urea cycle defects.

Prior studies in endothelial cells demonstrate that Arg2 enhances AKT (S473) phosphorylation independent of its hydrolytic activity. These studies asserted the mechanism to go through Arg2-dependent mTORC2 activation.^13^ In contrast, our unbiased transcriptomic data indicate that Arg2^H160F^ blocks both MTOR and AMPK, indicating cell-intrinsic signaling specificity in endothelial cells vs hepatocytes. Nevertheless, we extend prior Arg2-AKT regulation data to show that hepatocyte Arg2 enhances hepatic and peripheral AKT phosphorylation, increases peripheral energy expenditure, and suppresses hepatosteatosis independent of both hydrolytic activity and mitochondrial localization. Overall, we reveal enzymatic and localization-dependent- and independent Arg2 functions for Arg2 and demonstrate mitochondrial arginase activity may be a means to drive whole-body glucose oxidation and optimize glycemic control in the obese state.

## Methods

### Mice, Diets, and Treatments

All animal protocols were approved by the Washington University School of Medicine Animal Studies Committee. Male C57B/6J mice (Strain# 000664) and *db/db* mice (Strain# 000697) were purchased from the Jackson Laboratory and used for all experiments unless otherwise noted. Mice were randomized into treatment.

### Generation of Arg2^fl/fl^ and Arg2^fl/fl^ Alb-Cre Mice

The generation of mice carrying the Arg2 floxed allele was performed with the assistance of the Washington University in St Louis Genome Engineering & iPSC Center (GEiC) as previously described (Zhang et al., 2023, Submitted Manuscript). Briefly, LoxP sequences were inserted into the introns using CRISPR to flank the second exon of the wildtype *Arg2* allele on Chromosome 14 to generate mice harboring the conditional floxed *Arg2* allele. Offspring were subsequently crossed with wildtype C57B/6J mice and genotyped to confirm the presence of LoxP sites both upstream and downstream of exon 2 of the *Arg2* allele. To generate liver-specific *Arg2*^-/-^ knockout mice, Arg2 floxed mice (*Arg2*^fl/fl^ mice) were crossed with transgenic hemizygous C57B/6J mice expressing Cre under the Albumin promoter (Alb1-*Cre*, Strain# 016832) from Jackson Laboratory, which results in the recombination of the LoxP sites and removing exon 2, which is only 73 base pairs in length. The removal of exon 2 from the wildtype *Arg2* allele causes a frameshift mutation to trigger nonsense-mediated decay, which in turn creates hepatocyte-specific Arg2^−/−^ knockout mice.

All strains of genetically altered mice were on a C57BL/6J background. Control mice were littermates matched by genetic background, age, and sex in all experiments. All animals were housed at the Washington University Medical School in St Louis in a 12-hour alternating light-dark (lights on from 0600–1800), temperature-controlled, specific pathogen-free animal barrier facility prior to and throughout experimentation. Studies were conducted during the light cycle unless otherwise indicated. All animals were given ad libitum access to regular chow and sterilized water unless otherwise noted. All animal studies were performed in accordance with the criteria and ethical regulations outlined by the Institutional Animal Care and Use Committee (IACUC) under protocol number # 20-0330.

### AAV8-mediated Overexpression in vivo

AAV8 and adenovirus were administered via tail vein injection as previously reported.^2^ To increase liver transduction efficiency and liver specificity, AAV8, which has greater liver transduction efficiency, was used in combination with TBG promoter to maximize liver targeting specificity.^22^ 10^9^ particles per dose (adenovirus) and 10^11^ particles (AAV8) were delivered.^2^^,^^16^^,^^23^^,^^24^ All viral vectors were obtained directly from Vector Biolabs Inc.

### Indirect Calorimetry

All measurements were performed in a PhenoMaster System (TSE systems) via the Washington University Diabetic Mouse Models Phenotyping Core Facility. Mice were placed at room temperature (23–25 °C) in separate chambers of the PhenoMaster open-circuit calorimetry. Mice were acclimatized in the chambers for 48 to 72 hours prior to recording. For glucose oxidation measurements, food was removed, and mice were placed on aspen bedding. After 16 hours, each mouse received a mixture of 40 mg of [^13^C_6_]-labeled glucose and 130 mg of unlabeled glucose dissolved in 0.4 mL of 0.9% saline via intraperitoneal injection. Continuous measurements at an air sampling interval of 10 minutes were taken to allow the determination of glucose oxidation.

### Intraperitoneal Glucose Tolerance Test

Intraperitoneal glucose tolerance tests were carried out on mice fasted for 6 hours on aspen bedding. Basal blood glucose concentrations were determined for each mouse prior to glucose administration using a hand-held glucose meter (Arkray USA, Inc). Each mouse then received 2 g per kg body weight of glucose through intraperitoneal injection, and blood glucose concentrations were subsequently measured at 30, 60, 90, and 120 minutes post-glucose administration.

### Intraperitoneal Insulin Tolerance Test

Intraperitoneal ITTs were carried out on mice fasted for 4 hours on aspen bedding. Basal blood glucose concentrations were determined for each mouse prior to insulin administration using a hand-held glucose meter (Arkray USA, Inc). Each mouse then received 0.75 IU per kg body weight of insulin (Lilly USA, LLC) through intraperitoneal injection, and blood glucose concentrations were subsequently measured at 30, 60, 90, and 120 minutes post-insulin administration.

### Hyperinsulinemic-euglycemic Clamp

The hyperinsulinemic-euglycemic clamp study was conducted by the MMPC at Vanderbilt University. AAV8-TBG vectors encoding Arg2, Arg2^H160F^, Arg2^Δ1-22^, or GFP as the control were delivered into *db/db* mice via tail vein injection. Twenty-eight days post-AAV8 injection, hyperinsulinemic-euglycemic clamp was performed on 5-hour fasted mice. Insulin was continuously infused at a rate of 20 mU insulin per kg per minute. Continuous infusion of [6,6-^2^H_2_] glucose and ^2^H_2_O was done to assess glucose turnover rates and hepatic glucose fluxes.

### Clinical Chemistry Measurements and Serum Lipid Analyses

For all other serum analyses, submandibular blood collection was performed immediately prior to sacrifice, and serum was separated using BD Microtainer serum separators (BD, Ref# 365967) and frozen at −80 °C for storage. Serum lipid quantifications including serum TGs (Thermo Fisher Scientific Cat# TR22421), serum cholesterol (Thermo Fisher Scientific, Cat# TR13421), serum non-esterified free fatty acids (Wako Chemicals, #999-34691, #995-34791, #991-34891, and #993-35191), and serum low-density lipoprotein-cholesterol (Wako Chemicals, #993-00404, and #993-00504) were performed using commercially available reagents according to manufacturer’s directions. Serum ALT, aspartate aminotransferase, and albumin levels were quantified using an AMS Liasys Chemistry Analyzer (AMS Diagnostics, LLC). Serum ketone body levels were measured using a commercially available β-hydroxybutyrate Colorimetric Assay kit (Cayman Chemical, Cat# 700190). Serum insulin concentrations were measured using an Ultra-Sensitive Mouse Insulin ELISA kit (Crystal Chem, Cat# 90090). Serum glucose concentrations were measured using a glucose oxidase-based Glucose Colorimetric Assay kit (Cayman Chemical, Cat# 10009582). The HOMA-IR was calculated by dividing the results of the serum insulin concentrations (in μU/mL) and serum glucose concentrations (in mmol/L) by 22.5 for each individual animal.^25^

### Measurement of Liver Lipids

Liver-specific lipid concentrations were extracted and analyzed from snap-frozen liver tissue samples. Approximately 50-mg hepatic tissue samples were homogenized in 2:1 chloroform: methanol. In total, 0.25% to 0.5% of each extract was evaporated overnight prior to the biochemical quantification of triglycerides, cholesterol, and non-esterified free fatty acids using the reagents described above, precisely according to the manufacturer’s directions.

### Measurement of Liver Glycogen

Liver-specific glycogen concentrations were extracted and determined from snap-frozen liver tissue samples. Approximately 50-mg hepatic tissue samples were homogenized in 1 mL of assay buffer. Liver extracts were diluted 1:40 prior to colorimetric quantification of glycogen precisely according to the manufacturer’s directions (Cayman Chemical, Cat# 700480).

### Liver Histological Analysis

Formalin-fixed paraffin-embedded liver sections were stained with hematoxylin and eosin (H&E) via the Washington University Digestive Diseases Research Core Center (DDRCC) followed by microscopic examination for assessment of liver histology. Liver sections were stained with Sirius Red for assessment of liver fibrosis. OCT-embedded frozen liver sections were stained by Oil Red-O according to standard protocols.

### AML12 Mouse Hepatocyte Cell Culture

α mouse liver 12 (AML12) hepatocytes were purchased from the American Type Culture Collection (ATCC, Cat# CRL-2254; Research Resource Identifier [RRID]: CVCL_0140) and maintained per American Type Culture Collection guidelines.

### Primary Hepatocytes Isolation and Culturing ex vivo

All ex vivo primary hepatocytes were isolated and cultured from mice between the ages of 8- to 12-weeks old unless otherwise specified, as previously reported.

For experiments involving adenovirus-mediated overexpression, 1 × 10^8^ PFU per 1 mL of media per 1 well in a 6-well plate was added to the media to treat AML12 and 1 × 10^7^ PFU per 1 mL of media per 1 well to treat primary hepatocytes ex vivo. Adenovirus overexpression of mouse wildtype *Arg2*, mouse mutant *Arg2(H160F)*, mouse mutant *Arg2(d1-22AA)*, mouse wildtype *Arg1*, adenovirus overexpressing Cre, and the control adenovirus overexpressing eGFP were all obtained from Vector Biolabs. For experiments involving gene silencing through molecular approaches using either siRNA or ASO treatment, Lipofectamine RNAiMAX Transfection Reagent (Invitrogen, Cat# 13778150) was used per manufacturer’s instructions. Treated hepatocytes remained in culture for 48 hours prior to the next experimental step.

### Stable [U-13C]-Isotope Tracer Metabolomics

Primary hepatocytes were plated in 10-cm dishes and left undisturbed overnight at 37 °C. Then, [U-^13^C]-labeled medium was added to the primary hepatocytes and incubated for 24 hours before metabolite extraction. Both [U-^13^C] glucose and glutamine medium were made using a base DMEM media without glucose and glutamine (Gibco, Cat# A1443001). [U-^13^C_6_] glucose medium contained 25 mM labeled D-glucose (Cambridge Isotope Laboratories, Inc., Cat# CLM-1396) with 4.0 mM unlabeled L-glutamine (Sigma-Aldrich, Cat# G8540). [U-^13^C_5_] glutamine medium contained 25 mM unlabeled D-glucose (Sigma-Aldrich, Cat# G8270) with 4.0 mM labeled L-glutamine (Cambridge Isotope Laboratories, Inc., Cat# CLM-1822-H).

Methanol metabolite extraction was performed according to the HMT sample preparation protocol for metabolite extraction for adherent cells (protocol ver.ACB.1.0.0). Briefly, after 24 hours of incubation in labeling medium, the [U-^13^C] labeling culture medium was removed, and the primary hepatocytes were washed twice with 5% (w/v) D-mannitol (Sigma-Aldrich, Cat# M4125-500G) solution in Milli-Q water. The hepatocytes were then treated with 100% methanol (Fisher Scientific, Cat# A456-212) and homogenized for 30 seconds before Milli-Q water containing the HMT internal standards was added to the cell extract, followed by further homogenization for an additional 30 seconds. The cell extract was then centrifuged at 2,300 g at 4 °C for 5 minutes, after which the supernatant was centrifugally filtered at 4 °C through a 5-kDa cut-off filter (ULTRAFREE-MC-PLHCC, Human Metabolome Technologies) to remove macromolecules. The filtrate was evaporated under a vacuum to dry. The samples were stored at −80 °C until they were reconstituted and diluted in Milli-Q water for the downstream CE-MS metabolome analysis. The compounds were measured in the Cation and Anion modes of CE-TOFMS-based metabolome analysis. Data were corrected for the natural abundance of the stable isotope.

### AML12 Hepatocyte Ureagenesis Assay

At the start of the experiment, media was aspirated from 6-well plates and hepatocytes were washed 3 times with warm 1× phosphate buffered saline (PBS) (Gibco, Cat# 10010031). After the final wash, 1 mL of warm Hank’s balanced salt solution (HBSS) without glucose (formulation described below: Table 1) was added to each well and incubated for 3 hours at 37 °C. After incubation, HBSS without glucose was removed with an aspirator, and hepatocytes were washed once with fresh warm HBSS without glucose. After removing the wash solution, hepatocytes were treated with 1 mL of HBSS without glucose alone or with 20 mM of arginine for 4 hours at 37 °C. At the end of the incubation, the HBSS was collected and assayed for urea concentration to determine the rate of urea production using QuantiChrom Urea Assay Kit II (BioAssay Systems, Cat# DUR2-100). The hepatocytes are lysed in 1× RIPA buffer (Cell Signaling Technology, Cat# 9806) containing protease and phosphatase inhibitors (Thermo Scientific, Cat# A32961) on ice for 10 minutes with occasional diagonal agitation. BCA protein assay (Thermo Scientific, Cat# 23235) was performed to determine the protein concentration and was used to normalize urea production. The rate of ureagenesis was expressed as mg/dL urea/mg protein/hr.

**Table 1:** Formulation of HBSS Without Glucose

Components	Source	Identifier	Empirical formula	Formula weight, g/mol	Target concentration, mM	Required amount, mg
Inorganic salts						
Calcium chloride	Sigma-Aldrich	SKU C4901-100G	CaCl_2_	110.98	1.261	139.97
Magnesium chloride	Sigma-Aldrich	SKU M8266-100G	MgCl_2_-6H_2_O	203.30	0.493	100.15
Magnesium sulfate	Sigma-Aldrich	SKU M5921-500G	MgSO_4_-7H_2_O	246.47	0.407	100.19
Potassium chloride	Sigma-Aldrich	SKU P5405-250G	KCl	74.5513	5.333	397.61
Potassium phosphate monobasic	Thermo Scientific	Cat# 205925000	KH_2_PO_4_	136.086	0.441	60.04
Sodium bicarbonate	Thermo Scientific	Cat# BP328-1	NaHCO_3_	84.007	4.167	350.03
Sodium chloride	Sigma-Aldrich	SKU S9888-500G	NaCl	58.44	137.931	8060.69
Sodium phosphate dibasic anhydrous	Sigma-Aldrich	SKU S0876-100G	Na_2_HPO_4_	141.96	0.338	47.99
Other components						
HEPES	Sigma-Aldrich	SKU H3375-100G	C_8_H_18_N_2_O_4_S	238.3012	20.000	4766.02
Adjust PH to 7.4						
Sterile filter						

**Note:** The amounts listed for each component are for making 1 L of HBSS without glucose. pH is adjusted to 7.4 and filter-sterilized through a 0.2 μm vacuum filtration system (VWR, Cat# 10040-440). The final solution is kept at 4 °C and is stable for 3 months.

HBSS, Hank’s balanced salt solution.

### Extracellular Flux Analysis

In vitro respiration measurements were performed using the Seahorse xFE96 Analyzer (Agilent) with the AML12 immortalized mouse hepatocyte cell line as reported previously.^16^^,^^24^^,^^26^, ^27^, ^28^, ^29^, ^30^, ^31^

### Mitochondria Enrichment

Mitochondria were extracted using the Mitochondria Isolation Kit per the manufacturer’s instructions (Millipore Sigma, Cat# MIT0IS02). The amount of cytosolic and mitochondrial protein was quantified using a BCA assay prior to Western blotting.

### Quantitative Real-time RT-PCR

Total RNA was prepared by homogenizing snap-frozen livers or cultured hepatocytes in Trizol reagent (Invitrogen, Cat# 15596026) according to the manufacturer’s protocol. cDNA was synthesized using Qiagen QuantiTect Reverse Transcriptase kit (Qiagen, Cat# 205310). Real-time qPCR was performed with Step-One Plus Real-Time PCR System (Applied Biosystems) or QuantStudio 3 Real-Time PCR System (Thermo Scientific, Cat# A28567) using Fast SYBR Green Master Mix Reagent (Applied Biosystems, Cat# 4385612) and specific primer pairs (Table 2). Relative gene expression was calculated by a comparative method using values normalized to the expression of an internal control gene as indicated in the Figure legends.

**Table 2:** Mouse qRT-PCR Primer Sequences

Gene (mouse)	Forward (5' – 3')	Reverse (5' – 3')
*36B4*	TAA AGA CTG GAG ACA AGG TG	GTG TAC TCA GTC TCC ACA GA
*Arg2*	AGG AGT GGA ATA TGG TCC AGC	AGG GAT CAT CTT GTG GGA CAT T
*Lpl*	TCC GTG TCT GAC GAA GAA ATG	GCG GCC TTG AAC AAG TCA T
*Chrebp*	CTG GGG ACC TAA ACA GGA GC	GAA GCC ACC CTA TAG CTC CC
*Fasn*	CCT GGA TAG CAT TCC GAA CCT	AGC ACA TCT CGA AGG CTA CAC A
*Elvol6*	GAA AAG CAG TTC AAC GAG AAC G	AGA TGC CGA CCA CCA AAG ATA
*Acat1*	CAG GAA GTA AGA TGC CTG GAA C	TTC ACC CCC TTG GAT GAC ATT
*Acat2*	GGA CAG GGC ACC ATT GAA GG	CCC GTG GTC ATC GTC TCA G
*Gpam*	CAA CAC CAT CCC CGA CAT C	GTG ACC TTC GAT TAT GCG ATC A
*Pck1*	GAT GGG CAT ATC TGT GCT GG	CAG CCA CCC TTC CTC CTT AG
*β-Klotho*	TGT TCT GCT GCG AGC TGT TAC	TAC CGG ACT CAC GTA CTG TTT
*Bdh1*	GGT GGA ACC TGG CAA CTT CAT	GGT CAT CCC ACA TCT TCT TGG
*Hmgcr*	TCT GTT GTG AAC CAT GTG ACT TC	AGC TTG CCC GAA TTG TAT GTG
*Hmgcs1*	AAC TGG TGC AGA AAT CTC TAG C	GGT TGA ATA GCT CAG AAC TAG CC
*Il-1β*	GCA ACT GTT CCT GAA CTC AAC T	ATC TTT TGG GGT CCG TCA ACT
*Il-6*	TAG TCC TTC CTA CCC CAA TTT CC	TTG GTC CTT AGC CAC TCC TTC
*Tnf-α*	CAG GCG GTG CCT ATG TCT C	CGA TCA CCC CGA AGT TCA GTA G
*Ccl2*	TTA AAA ACC TGG ATC GGA ACC AA	GCA TTA GCT TCA GAT TTA CGG GT
*Cxcl9*	GGA GTT CGA GGA ACC CTA GTG	GGG ATT TGT AGT GGA TCG TGC

qRT-PCR, Real-time quantitative reverse transcription polymerase chain reaction.

### Two-photon Microscopy

mito::*mKate*2 mice (Jax, Stock # 032188) were transfected with adenovirus expressing either eGFP, Arg2, Arg2(H160F), or Arg2 (d22AA) via tail vein injections. Two weeks post-transfection, mice were anesthetized with isoflurane gas and subsequently euthanized. The livers were explanted, glued onto a plastic coverslip, and secured using vacuum grease in a petri dish containing Co2-independent media. The livers were imaged with an excitation wavelength of 900 nm with emission filters of 495nm, 540 nm, and 605 nm. To document localization and colocalization of the proteins, images were acquired in the following parameters: 1024 × 1024 pixels, 0.585 pixels/um, 10f average/z.

### Untargeted Metabolomics Using Ultrahigh Performance Liquid Chromatography-tandem Mass Spectroscopy

Untargeted metabolomics was performed by Metabolon, Inc. Samples were prepared using the automated MicroLab STAR system from Hamilton Company. Several recovery standards were added prior to the first step in the extraction process for quality control purposes. To remove protein, dissociate small molecules bound to protein or trapped in the precipitated protein matrix, and to recover chemically diverse metabolites, proteins were precipitated with methanol under vigorous shaking for 2 minutes (Glen Mills GenoGrinder 2000) followed by centrifugation. The resulting extract was divided into 5 fractions: 2 for analysis by 2 separate reverse phase (RP)/ ultrahigh performance liquid chromatography-tandem mass spectroscopy (UPLC-MS/MS) methods with positive ion mode electrospray ionization (ESI), one for analysis by RP/UPLC-MS/MS with negative ion mode ESI, one for analysis by HILIC/UPLC-MS/MS with negative ion mode ESI, and one sample was reserved for backup. Samples were placed briefly on a TurboVap (Zymark) to remove the organic solvent. The sample extracts were stored overnight under nitrogen before preparation for analysis. The MS analysis alternated between MS and data-dependent MSn scans using dynamic exclusion. The scan range varied slightly between methods but covered 70 to 1000 m/z. Raw data was extracted, peak-identified, and quality control processed using Metabolon’s hardware and software.

### Immunoblotting

Tissues were homogenized in RIPA buffer supplemented with protease and phosphatase inhibitors (Thermo Scientific). After homogenization, lysates were centrifuged at 18,000g for 15 minutes at 4 °C, and the supernatant was recovered. Protein concentration was determined by BCA Assay Kit (Thermo Scientific) and was adjusted to 2 mg/mL. Samples for Western blotting were prepared by adding 2× Laemmli sample buffer (Bio-Rad, Cat# 1610737EDU) at a ratio of 1:1 and heating at 95 °C for 5 minutes. The prepared samples were subjected to 10% or 13% SDS-PAGE, followed by electrical transfer onto a nitrocellulose membrane using the Trans-Blot Turbo system (Bio-Rad). After blocking the membrane with 5% milk in TBST, the membrane was incubated in primary antibody at 4 °C overnight. The blot was developed after secondary antibody incubation using Pierce ECL Western Blotting Substrate (Thermo Scientific). Blots were developed according to the manufacturer’s instructions. Protein expression levels were quantified with Image Lab software and normalized to the levels of β-Actin, Vinculin, or GAPDH.

### Antibodies

Antibodies against GFP (Cat# 2956), Cytochrome C (CYCS, Cat# 4280), phospho-AKT (Ser473) (Cat# 9271), AKT (Cat# 9272), GAPDH (Cat# 5174), Vinculin (Cat# 13901), and β-ACTIN (Cat# 3700) were purchased from Cell Signaling Technology. The dilution ratio for all primary antibodies was 1:1,000. The secondary antibodies used in this study were peroxidase-conjugated anti-rabbit IgG (Cat# 7074) and anti-mouse IgG (Cat# 7076) purchased from Cell Signaling Technology, which were used at a 1:5,000 dilution.

### RNA-sequencing

RNA-sequencing was performed by the Washington University Genome Technology Access Center as described.^17^^,^^23^^,^^32^

### Statistical Analyses

Data were analyzed using GraphPad Prism version 9.5.1 (RRID:SCR_002798). Data shown are as mean ± standard error of the mean unless specified otherwise. *P* ≤ .05 was defined as statistically significant. Statistical comparisons were made using unpaired 2-tailed homoscedastic Student t-tests or analysis of variance for analyses with 2 independent variables with Dunnett’s post hoc correction for multiple comparisons where appropriate for all analyses unless otherwise noted in the figure legends.
